# Environment and Offspring Surveillance in Porcine Brucellosis

**DOI:** 10.3389/fvets.2022.915692

**Published:** 2022-06-21

**Authors:** Agustín Rebollada-Merino, Marta Pérez-Sancho, Antonio Rodríguez-Bertos, Nerea García, Irene Martínez, Alejandro Navarro, Lucas Domínguez, Teresa García-Seco

**Affiliations:** ^1^VISAVET Health Surveillance Centre, Complutense University of Madrid, Madrid, Spain; ^2^Department of Internal Medicine and Animal Surgery, Faculty of Veterinary Medicine, Complutense University of Madrid, Madrid, Spain; ^3^Department of Animal Health, Faculty of Veterinary Medicine, Complutense University of Madrid, Madrid, Spain

**Keywords:** *Brucella suis*, brucellin skin test, environment, post-weaning pigs, surveillance

## Abstract

Porcine brucellosis, caused by *Brucella suis* (*B. suis*), is a notifiable disease causing significant economic losses in production systems. Most infected pigs may act as carriers and shed *B. suis* even if asymptomatic. This can contribute to environmental persistence, thus hindering control efforts. Here, the environment and the offspring were investigated during and after a *B. suis* outbreak at a sow breeding farm. The diagnosis of *B. suis* in sows (*n* = 1,140) was performed by culture and polymerase chain reaction (PCR) from vaginal swabs, indirect enzyme-linked immunosorbent assay (I-ELISA) from sera, and brucellin skin test (BST). *B. suis* diagnosis in post-weaning pigs (*n* = 899) was performed by I-ELISA in sera and BST. The environmental surveillance programme was implemented by placing gauze sponges (*n* = 175) pre-hydrated in a surfactant and inactivating liquid for *Brucella* DNA detection by PCR in different farm areas. Our results showed that the offspring of infected sows reacted to *in vivo* techniques for *B. suis*. Furthermore, the offspring born during the outbreak displayed higher seropositivity (I-ELISA) and reactivity (BST) than those pigs born after. *Brucella* DNA was detected in pregnant sow areas, boxes, boots, and post-weaning pig areas. In addition, *Brucella* DNA environmental detection was higher during the *B. suis* outbreak than the post *B. suis* outbreak. The environmental approach has proven to be a simple, practical, valuable, and safe method to detect and monitor *B. suis*. These results suggest a role of the environment and the offspring that should be considered in porcine brucellosis surveillance and control programmes.

## Introduction

Brucellosis [*Brucella abortus (B. abortus), B. melitensis, B. suis*] is a notifiable disease according to the World Organization for Animal Health (OIE) (1). Porcine brucellosis is a worldwide-distributed, re-emerging disease caused by *B. suis* biovars 1, 2, and 3, of which biovar 2 is the most prevalent in domestic swine in Europe (2). *B. suis* surveillance is mandatory in insemination centers and during exports–imports in the European Union (EU) [Commission Delegated Regulation (EU) 2020/688 of 17 December 2019].

Definitive diagnosis is achieved by bacterial culture and isolation followed by polymerase chain reaction (PCR) confirmation (1), which is a time-consuming approach, limited by laboratory resources, and with a variable sensitivity (3). *In vivo* diagnosis in domestic swine relies on humoral-based and/or cellular-based techniques. Serological assays comprise the buffered *Brucella* antigen tests, the complement fixation test, the indirect enzyme-linked immunosorbent assay (I-ELISA), the fluorescence polarization assay, and the competitive enzyme-linked immunosorbent assay. The brucellin skin test (BST) is a cellular-based assay founded on the delayed type IV hypersensitivity reaction to cytosolic and periplasmatic protein extracts inoculated in the skin (4, 5). The complementary use of I-ELISA and BST increases diagnostic accuracy (3, 6).

*Brucella suis* infection leads to significant economic losses (7). *B. suis* can cause infertility and reproductive failure at any moment during pregnancy, mainly in the last third (8, 9). Shedding occurs *via* semen, uterine/vaginal discharges, placenta, and tissues from abortions/dead piglets, as well as in urine and milk, and it is transmitted *via* direct contact with mucous membranes (mating, perinatal, and throughout ingestion of milk by piglets or aborted remains by sows) (10, 11). However, the pathogenesis and epidemiology of brucellosis in swine are not widely characterized. Most infected pigs may act as asymptomatic carriers and shedders, contributing to the maintenance and spread of the disease in the herd due to the ability of *B. suis* to survive in the environment (11, 12). Despite this, the role of the environment in the epidemiology of porcine brucellosis is yet to be ascertained.

Porcine brucellosis pre-movement surveillance programme in the EU lays down that the pigs must come from a farm with no cases of brucellosis during the 42 days prior to departure, and where for at least 12 months prior to departure the pigs have been subjected to surveillance for brucellosis using immunological assays demonstrating the absence of brucellosis at a target prevalence of 10% (EU Commission Delegated Regulation 2020/688 of 17 December 2019). Despite the fact that the use of single or multiple diagnostic techniques in sows and boars has been widely studied, there is a lack of research published on the use of diagnostic techniques in young pigs. Thus, the humoral- and cellular-based immune responses in offspring born from *B. suis*-infected sows are still unknown.

Herein, we present the results of research focused on the environment and offspring during and after a *B. suis* outbreak at a sow breeding farm, which, to the best of the authors' knowledge, has not been previously evaluated in the context of porcine brucellosis. We sought to research the environmental bacterial DNA distribution and persistence as the control measures take place using *B. suis*-inactivating surfactant-hydrated sponges that allowed DNA detection. By monitoring post-weaning pigs, we aimed to assess the reactivity to commonly-employed diagnostic techniques and the correlation with the diagnostic results obtained in sows.

## Materials and Methods

The diagnostic procedures were carried out at a sow breeding farm (*n* = 500) with an open production system for a two-year period. Part of the post-weaned pigs was raised for up to 2 months (*n* = 3,000). The replacement rate was 60%, and the percentage of abortions historically did not exceed 2%. From January 2016 onwards, a gradual rise in reproductive failures, especially in late-term abortions up to 6%, raised suspicions of a reproductive problem compatible with *B. suis* infection.

### *In vivo* Assays for *B. suis* Diagnosis in Sows

*Brucella suis* diagnosis in sows was performed by culture, isolation, and PCR confirmation in vaginal swabs; I-ELISA and BST, in accordance with the Manual of Diagnostic Tests and Vaccines for Terrestrial Animals (1). The diagnosis was grouped in rounds of 3 months to screen the highest number of sows. Therefore, 35% of the farm census (*n* = 84) was screened during the period the abortions lasted (3 months—first round), and 100% of the farm census was screened twice after the last *B. suis*-abortion (*n* = 468, from months 1–5 after the last abortions—second round, and *n* = 588, from months 6–10 after abortions—third round).

Samples of vaginal swabs (*n* = 1,140) of sows displaying reproductive failures (including abortions), and also vaginal swabs from sows without reproductive failures (1-week post-delivery), were collected in Amies transport medium (Deltalab, Barcelona, Spain). The vaginal swabs were cultured, and DNA was extracted using a commercial extraction kit (MagMAX CORE Nucleic Acid Purification Kit, Applied Biosystems, Foster City, CA) and an automated extraction robot (KingFisher Flex, Thermo Fisher Scientific). *Brucella* detection was performed using a previously described PCR protocol (13). A commercial multiplex conventional PCR was used for the identification of *B. suis* biovars 1 to 5 (INgene Bruce-ladder Suis, Ingenasa, Madrid; Spain). There were negative results by specific PCR techniques after direct extraction of DNA/RNA for swine abortive agents such as porcine reproductive and respiratory syndrome virus (14), porcine herpesvirus types 1 and 2 (15), porcine circovirus type 2 (16), porcine parvovirus type 1 (17), *Leptospira interrogans* (18), *Chlamydia suis* (19), and *Toxoplasma gondii* (20).

To determine the presence of antibodies against *Brucella*, blood samples were collected. Sera were tested with a commercial I-ELISA kit that detects IgG against *Brucella* lipopolysaccharide (LPS) (Ingezim *Brucella* Porcina, Ingenasa, Madrid, Spain). Results were interpreted according to the manufacturer's instructions.

To assess the cellular immune response to *Brucella*, 0.1 ml of a commercial antigen (Brucellergene OCB, Zoetis, Parsippany-Troy Hills, NJ) was inoculated intradermically in the base of the tail, as described previously (3, 4). A reaction in the inoculation site 48 h post-inoculation, associated with a delayed-type IV hypersensitivity response, was considered positive if inflammation or hemorrhage was present.

### *In vivo* Assays for *B. suis* Diagnosis in Post-Weaning Pigs

In 2-month-old post-weaned pigs, I-ELISA and BST were performed monthly at seven moments in time: four consecutive samplings in weaned pigs born during the time the abortions lasted and three consecutive samplings in pigs born after the last *B. suis*-abortion.

To determine the presence of antibodies against *Brucella*, blood samples were collected. Sera were tested with a commercial I-ELISA kit that detects IgG against *Brucella* LPS (Ingezim *Brucella* Porcina, Ingenasa, Madrid, Spain). Results were interpreted according to the manufacturer's instructions.

To assess the cellular immune response to *Brucella*, 0.1 ml of a commercial antigen (Brucellergene OCB, Zoetis, Parsippany-Troy Hills, NJ) was inoculated intradermically in the base of the tail, as described previously (3, 4). A reaction in the inoculation site 48 h post-inoculation, associated with a delayed-type IV hypersensitivity response, was considered positive if inflammation or hemorrhage was present.

### *In vitro* Assays for the Validation of *B. suis* Inactivation and Conservation of Bacterial DNA in a New Surfactant Isotonic Liquid for Environmental Samplings

The new surfactant liquid designed for environmental sampling (Spanish patent, number P2115ES00) was obtained by mixing equal parts of solution 1 (isopropyl alcohol 99.8%, ethanol 99.8%, methanol 99.9%, and glycerol) and solution 2 (disodium phosphate, sodium dodecyl sulfate 0.1%, and nuclease-free water). This surfactant liquid has proven to inactivate microorganisms of animal and public health importance as well as to preserve their genetic material for molecular detection tests (21–23).

First, a test was carried out to confirm the surfactant isotonic liquid to inactivate *B. suis*. Briefly, *B. suis* colony growth in purity was suspended in a 0.5 McFarland 0.85% sterile saline solution (SS). Afterward, 1 ml of the suspension was dispensed into a tube with 9 ml of sterile 0.85% SS (tube A, viability and purity control, 10^7^ CFU/ml *B. suis* expected concentration) and two tubes with 9 ml of the surfactant isotonic liquid (tubes B and C). The suspensions were homogenized by vortexing, and the incubation was performed at three moments in time: 10 min, 1, and 24 h. After each of the times, 100 μl of the mass suspension in solid medium Agar Columbia was seeded in each tube in order to evaluate *B. suis* inactivation. The seeding of each tube at each time was performed in duplicate. In addition, in the case of viability and purity control tube, serial dilutions in phosphate-buffered saline (PBS) were performed on a 1:10 basis to estimate the concentration of the inoculum. Incubation of the plates was carried out in aerobiosis at 37°C for 24 h. After incubation, the plates were read to check whether or not there was bacterial growth. In order to evaluate *B. suis* DNA preservation in the sampling liquid, samples from tubes A, B, and C after 24 h of incubation were subjected to DNA extraction, purification, and *B. suis* DNA detection by PCR in accordance with the protocols described in “DNA extraction and real time polymerase chain reaction (PCR)” section.

The performance of the surfactant liquid was compared with buffered peptone water (BPW). BPW is a culture and transport medium commonly used in surface and carcass sampling for isolation and detection of bacterial species such as *Salmonella* and *Listeria*. By doing so, once the *B. suis* outbreak was confirmed, a preliminary environmental sampling was performed on the farm using the surfactant liquid and BPW in parallel.

### *B. suis* DNA Detection in Environmental Samples

To monitor the environmental presence of *Brucella*, Dry Sponges 3 M (3 M Dry-Sponge; 3 M, Madrid, Spain) were pre-hydrated in the previously cited surfactant and pathogen-inactivating isotonic liquid (15 ml/sponge).

The sponges were randomly placed around the farm facilities in different locations: boxes, pregnant sow areas, post-weaning pig areas, and boots (Table 1). Seven samplings were performed: three consecutive samplings during the time the abortions lasted, and four samplings after the last *B. suis*-abortion. After sampling, the sponges were preserved at room temperature in a plastic bag ensuring bio-safety.

**Table 1:** Results for the polymerase chain reaction (PCR) evaluation of environmental samples at different moments in time (months) and in different locations throughout the study.

	**During** ***B. suis*** **abortions**			**After** ***B. suis*** **abortions**					
	**M1**	**M2**	**M3**	**M6**	**M9**	**M12**	**M24**	**Total**	**%**
Boxes	3/7	6/9	5/7	0/11	4/11	0/8	0/11	18/64	28.1
Pregnant sow areas	6/6	4/11	7/9	0/11	1/7	4/13	0/7	22/64	34.4
Post-Weaning pig area	2/3	0/2	2/6	0/2	0/7	0/12	0/7	4/39	10.3
Boots	0/0	1/1	1/1	0/2	0/2	0/2	0/0	2/8	25.0
Positive samples	11/16	11/23	15/23	0/26	5/27	4/35	0/25	46/175	26.3
%	68.8	47.8	65.2	0.0	18.5	11.4	0.0	26.3	

Environmental samples (from *in vitro* inactivation assays, from the preliminary assay comparing the surfactant liquid and BPW, and from the environmental sponges embedded in the surfactant liquid) were extracted using a commercial extraction kit (MagMAX CORE Nucleic Acid Purification Kit, Applied Biosystems, Foster City, CA) and an automated extraction robot (KingFisher Flex, Thermo Fisher Scientific). *Brucella* detection was performed using a previously described PCR protocol (13).

### Statistical Analysis

All statistical analyses were performed using IBM SPSS Statistics Software v. 25 (IBM; Armonk, NY, USA). Multiple comparisons of proportions were estimated using Z-test with a Bonferroni adjustment. Comparisons of two proportions were estimated by using Fisher's test. The level of significance was set at *p* < 0.05. Concordance between diagnostic techniques was evaluated by Cohen's kappa coefficient, according to the following interpretation: 0.0–0.2 insignificant, 0.2–0.4 low, 0.4–0.6 moderate, 0.6–0.8 good, and 0.8–1.0 very good.

## Results

*Brucella suis* biovar 2 was diagnosed as the cause of abortions. Consequently, the farm's veterinary staff implemented a control programme over a 2-year period based on a test culling strategy in sows and post-weaning pigs.

### *B. suis* Diagnosis in Sows

Most vaginal swabs recovered from aborted sows were positive for *Brucella* (69/84, 82.1%) during the first round (*B. suis*-abortions) of PCR testing. Vaginal swabs taken during the second and third rounds (after the last *B. suis*-abortion) were negative for *B. suis*.

Reproductive failures associated with *B. suis* occurred mainly during the last third of gestation (37/84, 44.0%), while the first (23/84, 27.4%) and second (24/84, 28.6%) thirds were equally represented. The distribution of the abortions per productive cycle showed an increase in the cases in primiparous sows (25/84, 29.8%) and sows in their second (20/84, 23.8%) and third (14/84, 16.7%) cycles. Abortions were minimal in sows of the fourth (6/84, 7.1%), fifth (7/84, 8.3%), sixth (9/84, 10.7%), and seventh cycles (3/84, 3.6%).

The percentage of I-ELISA-positive sows showed a statistically significant decrease in each round with regard to the previous one: 92.3% (180/195) in the first, 79.7% (373/468) in the second, and 30.4% (179/588) in the third rounds (*p* < 0.05).

For BST, positive reactions were found in 51.1% (285/558) in the first, 78.6% (368/468) in the second, and 34.3% (202/588) in the third rounds, with statistically significant differences between phases (*p* < 0.05).

### *B. suis* Diagnosis in Post-Weaning Pigs

**Figure 1:**
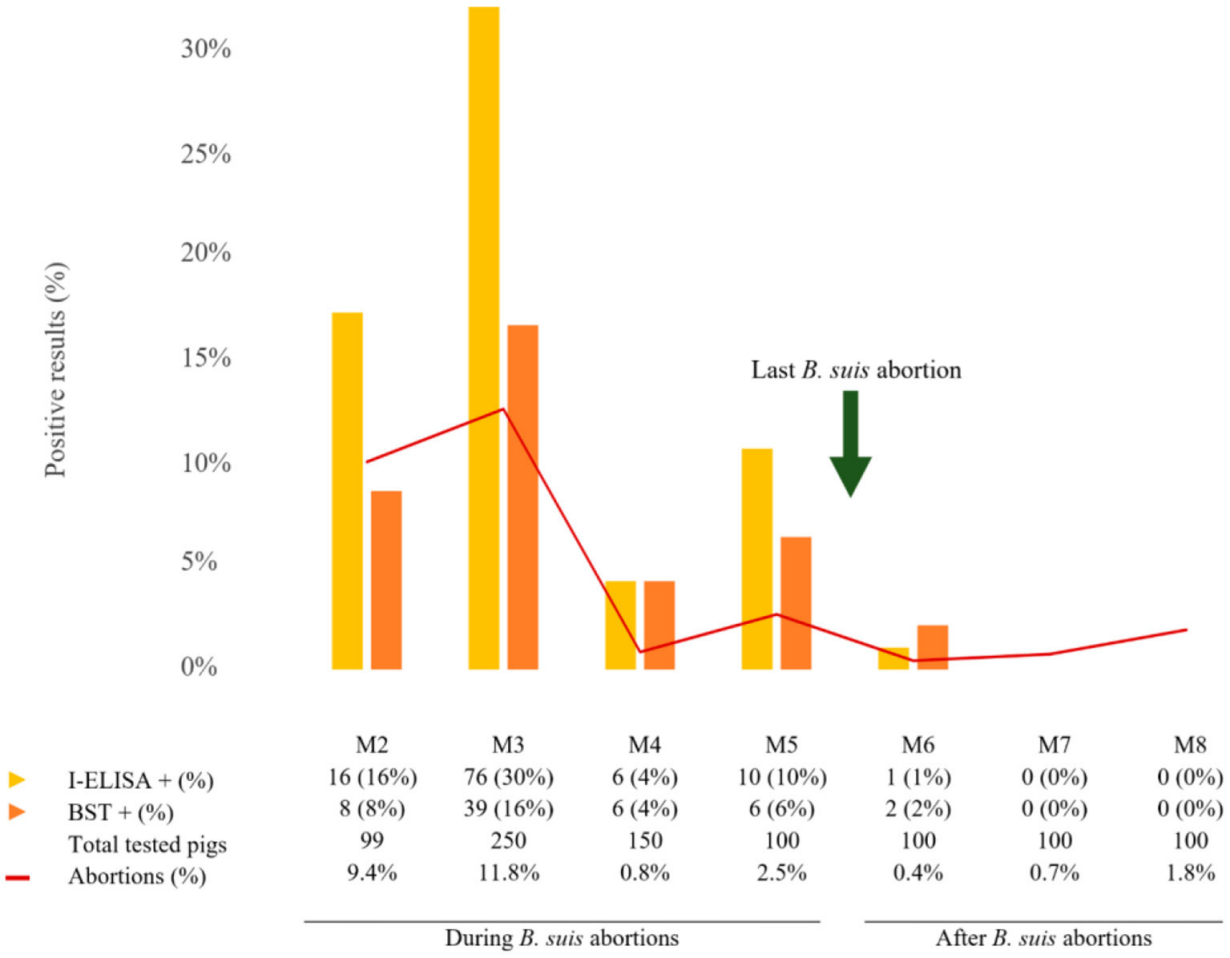
Histogram representing the number of post-weaning pigs, positive or negative to indirect enzyme-linked immunosorbent assay (I-ELISA, yellow) and brucellin skin test (BST, orange), related to total abortions registered in the farm (red line). Results are grouped in pigs born during and after *B. suis* abortions.

The results of I-ELISA showed that pigs born during *B. suis* abortions displayed higher seropositivity rates (4–30%) than after (0–1%) (Figure 1).

The BST results showed increased rates of positive reactors during (4–16%) than after *B. suis* abortions (0–2%) (Figure 1). The positive reactor proportion was lower than seropositive pigs throughout the study.

The concordance between both diagnostic techniques was moderate (Cohen's kappa coefficient = 0.522).

### *B. suis* Inactivation and DNA Preservation Using Environmental Sponges

The total inactivation of *B. suis* after 10 min of contact with the surfactant isotonic liquid was demonstrated, according to the total absence of colonies observed when samples after 10 min, 1 h, and 24 h of contact with the surfactant liquid were cultured in agar Columbia medium, contrasting with a growth corresponding with 10^7^ UFC/ml of control *B. suis* inoculum re-suspended in 0.85% SS.

Furthermore, PCR results obtained after the duplicate extraction of both tubes inoculated with *B. suis* in the surfactant liquid and the control tube (*B. suis* in 0.85% of SS) were equivalent, amplifying at cycles 26–27 in all cases.

The preliminary assay comparing the surfactant liquid and BPW revealed a total of 20 PCR-positive samples (20/26, 76.9%): 14 positives with both methods, 4 positives with the surfactant liquid alone, and 2 positives with BPW alone.

### *Brucella* DNA Detection in the Environment

*Brucella* DNA was detected in boxes (18/64, 28.1%), pregnant sow areas (22/64, 34.4%), post-weaning pig areas (4/39, 10.3%), and boots (2/8, 25.0%) (Table 1).

Furthermore, *Brucella* DNA environmental detection was higher during (47.2–68.8%) than after *B. suis* abortions (0–18.5%) (Table 1).

## Discussion

The presence of *B. suis* in the environment may contribute to the transmission of the disease on farms (8, 12). However, there are few baseline data demonstrating that the environment may represent a component contributing to transmission. Here, the presence of environmental *B. suis* DNA was determined during and after the outbreak. To the authors' knowledge, this is the first study describing environmental surveillance and *B. suis* DNA presence at porcine farm facilities.

*Brucella* culture is challenging, and it is difficult to isolate from environmental samples (24). To solve this, we pre-hydrated gauze sponges in the isotonic surfactant and pathogen-inactivating liquid described to simplify application and enhance bio-safety during transport and sample handling while preserving the DNA. The detection of *B. suis* DNA indicates the usefulness of this sampling method in brucellosis environmental surveillance. Despite the fact that DNA detection does not allow viable bacteria to be distinguished (25), environmental DNA detection provides valuable information in the context of surveillance programmes in swine (26). In this context, environmental sampling could be a useful tool for assessing *B. suis* distribution and persistence.

The detection of bacterial or viral nucleic acids using the same pre-hydrated sponge approach has been successfully applied in the environmental detection of such notifiable pathogens as *Mycobacterium tuberculosis* complex (23), *Mycobacterium avium* complex (unpublished data), SARS-CoV-2 (21), and African swine fever virus (22). Environmental sampling using methods other than sponges has already proven useful for the detection of slow cultured bacteria such as *Mycobacterium tuberculosis* complex (27) and *Mycoplasma hyopneumoniae* (26), and abortive agents such as *Chlamydia suis* (28) and *Coxiella burnetii* (29, 30). Environmental detection of *Brucella* microti-like during an outbreak at a frog farm has also been described (31).

The initial results observed here indicate that *B. suis* could be detected in the environment, where it may be shed by infected pigs and may be associated with the high prevalence observed. It also constitutes a potential focus of indirect infection to naïve pigs if cleaning and disinfection measures are not properly emphasized. Thus, positive environmental samples were detected months after the last confirmed *B. suis* abortion, during periods without detection of PCR-positive vaginal swabs, even in locations subject to cleaning and disinfection procedures. Environmental sampling could be a complementary (or even alternative) and non-invasive and safe technique to animal testing in routine screenings in closed loop productions (32). Moreover, unlike vaginal swabs, which are only useful during or shortly after parturition, environmental samples can be used throughout the production cycle, especially in farm zones areas where non-pregnant pigs are raised (25). In fact, *B. suis* DNA was detected in the post-weaning areas, suggesting that post-weaning pigs may excrete into the environment and may thus contribute to porcine brucellosis perpetuation and the risk of their spread to other farms.

Environmental presence of *Brucella* DNA progresivelly decreased up to a complete absence 2 years after the outbreak. These results suggested that *B. suis* was permanently shed until control measures were implemented, and/or that the cleaning and disinfection procedures were not effective in totally removing environmental *B. suis*. In fact, positive environmental samples continued to be observed long after the last *Brucella*-associated abortion was detected, so it can be assumed that even if outbreaks are controlled, the pigs may remain at risk of infection for a long period of time if all potential shedders are not diagnosed and sent to slaughter.

We assume that the environmental reduction of DNA *Brucella* was provided by a reduction in the potential shedders and by an improvement in the cleaning and disinfection protocols and bio-safety. The results obtained in the present study support the need to implement new control measures for porcine brucellosis on farms. Specifically, the use of environmental sampling has proven to be a simple, practical, valuable, and safe method to detect and monitor *B. suis* DNA persistence at farm facilities, and a suitable system to implement in order to evaluate the efficacy of *B. suis* control programmes on farms. Our results also highlight the need to carry out successive sampling after a negative result to ensure negative environmental presence, as this may be subject to variations due to different factors (such as shedders and non-sampled areas). Further, controlled experimental studies are necessary to ascertain the role of the environment on *B. suis* transmission by means of placing naïve pigs into a *Brucella*-contaminated environment.

Herein, we also aimed to compare how weaned pigs, born in the context of a clinical brucellosis outbreak, reacted to diagnostic techniques in periods with and without abortions caused by *B. suis*, which, to our knowledge, has not been previously reported. This may be useful to understand the epidemiology of *B. suis* during outbreaks and the specific roles of post-weaning pigs in porcine brucellosis aside from the immunological response. Our results show that pigs born during the period of time that abortions lasted display higher seropositivity and reactivity than those born after the last *B. suis*-confirmed abortion (30 vs. 1% positivity for I-ELISA and 16 vs. 2% for BST), suggesting that the immune response of post-weaned pigs correlates with the epidemiological scenario on the farm. The progressive decrease in on-farm infection pressure due to the measures implemented may have prevented both the vertical and horizontal infection of piglets from a certain point in time onwards.

Also, we have observed a reduced percentage of seropositive post-weaning pigs compared with sows. This coincides with previous studies that observed a lower number of seropositive in the progeny compared with females in buffalo (33, 34), bison (35), and domestic cattle (36) infected with *Brucella*. We found that post-weaning pigs displayed a cellular response to *Brucella* antigens by means of BST, which to our knowledge has not been evaluated before in the offspring of females infected by any *Brucella*. The use of BST in post-weaning pigs may help to truly distinguish infections from “false” seropositive piglets due to colostrum intake within the first month of life (37).

The combination of humoral-based and cellular-based diagnostic assays in the context of porcine brucellosis allows us to avoid correlation errors as each technique is biologically independent (2). Here, the moderate concordance (Cohen's kappa coefficient = 0.522) between both tests in post-weaning pigs shows the usefulness of employing both techniques in parallel in order to increase the detection of positive pigs. Concordance is widely variable depending on the epidemiological context, as previously described (38). This study suggests that a representative sampling of post-weaning pigs could serve as an indirect indicator of *B. suis* infection in sows. This approach could be useful in the national and international commercial trade of young pigs, as the early detection of infected pigs that may act as potential shedders reduces the risk of *B. suis* dissemination (8, 11).

## Data Availability Statement

The original contributions presented in the study are included in the article/supplementary material, further inquiries can be directed to the corresponding author/s.

## Ethics Statement

Ethical review and approval was not required for the animal study because the samples were obtained from routinary diagnostic procedures in a porcine farm.

## Author Contributions

MP-S, LD, and TG-S: conceptualization. AR-M, AR-B, NG, IM, and AN: methodology. AR-M and TG-S: software and formal analysis. MP-S, AR-B, NG, IM, LD, and TG-S: validation. AR-M, MP-S, AR-B, NG, IM, AN, and TG-S: investigation. LD: resources and visualization. AR-M: writing—original draft preparation. MP-S and TG-S: writing—review and editing. AR-B, LD, and TG-S: supervision. AR-B and LD: funding acquisition. All authors contributed to the article and approved the submitted version.

## Funding

This research was partially funded by the Spanish Ministry of Science and Innovation and the Spanish Ministry of Universities (RTI-2018/098658-B-C22) as AR-M was a recipient of a Spanish government-funded Ph.D. contract for research staff training (FPI).

## Conflict of Interest

The authors declare that the research was conducted in the absence of any commercial or financial relationships that could be construed as a potential conflict of interest.

## Publisher's Note

All claims expressed in this article are solely those of the authors and do not necessarily represent those of their affiliated organizations, or those of the publisher, the editors and the reviewers. Any product that may be evaluated in this article, or claim that may be made by its manufacturer, is not guaranteed or endorsed by the publisher.
